# Development of a New Deodorization Method of Herring Milt Hydrolysate: Impacts of pH, Stirring with Nitrogen and Deaerator Treatment on the Odorous Content

**DOI:** 10.3390/foods10040884

**Published:** 2021-04-17

**Authors:** Sarah Todeschini, Véronique Perreault, Charles Goulet, Mélanie Bouchard, Pascal Dubé, Yvan Boutin, Laurent Bazinet

**Affiliations:** 1 Department of Food Sciences and Laboratoire de Transformation Alimentaire et Procédés ÉlectroMembranaires (LTAPEM, Laboratory of Food Processing and ElectroMembrane Processes), Université Laval, Québec, QC G1V 0A6, Canada; sarah.todeschini.1@ulaval.ca (S.T.); veronique.perreault.5@ulaval.ca (V.P.); 2 Institute of Nutrition and Functional Foods (INAF), Université Laval, Québec, QC G1V 0A6, Canada; yvan.boutin@tbt.qc.ca; 3 Department of Phytology, Université Laval, Québec, QC G1V 0A6, Canada; charles.goulet@fsaa.ulaval.ca; 4 Investissement Québec-Centre de Recherche Industrielle du Québec (CRIQ, Quebec Investment–Industrial Research Center of Quebec), Québec, QC G1P 4C7, Canada; melanie.bouchard@invest-quebec.com (M.B.); pascal.dube@invest-quebec.com (P.D.); 5 Centre Collégial de Transfert de Technologie en Biotechnologie (TransBIOTech, College Center for Technology Transfer in Biotechnology), Lévis, QC G6V 6Z9, Canada

**Keywords:** herring milt hydrolysate, deodorization, deaerator, off-flavors, trimethylamine, dimethylamine, trimethylamine oxide, sensory analysis

## Abstract

Herring milt hydrolysate (HMH) presents the disadvantage of being associated with an unpleasant smell limiting its use. Thus, to develop a new effective and easy-to-use deodorization method, this research aimed to deepen the knowledge regarding the impacts of pH (pH 7 vs. pH 10), overnight stirring with nitrogen (+N vs. −N) and deaerator treatment (+D vs. −D) on the odorous content of HMH. This latter included dimethylamine (DMA), trimethylamine (TMA), trimethylamine oxide (TMAO) and the most potent odor-active compounds of HMH. Results showed that pH had a huge impact on the targeted compounds resulting in higher detected concentrations of DMA, TMA and TMAO at pH 10 than at pH 7 (*p* < 0.05) while the opposite trend was observed for the most potent odor-active compounds of HMH (*p* < 0.05). Moreover, independently of the pH condition, the overnight stirring with or without nitrogen had no impact (*p* > 0.05). Finally, the deaerator treatment was more effective to remove TMA and DMA at pH 10 than at pH 7 (*p* < 0.05) while the opposite trend was observed for the most potent odor-active compounds (*p* < 0.05). Sensory analysis confirmed that the application of pH 10 −N +D and pH 7 −N +D + alkalization pH 10 conditions led to the least odorous products (*p* < 0.05).

## 1. Introduction

Due to environmental and economic concerns, more and more attention has been paid to the valorization of by-products originating from fish processing plant. These by-products are generally composed of skin, head, bones, viscera, trimmings, milt and could represent from 50 to 70% of the fish fresh weight with regards to the involved species [1]. More specifically, concerning fish milt, this by-product was treated as a waste for a long time resulting in its underutilization [2]. Nevertheless, its content in added-value compounds such as proteins, polyunsaturated fatty acids, nucleic acids and vitamins makes it, nowadays, an interesting renewable bioresource that is worth to better consider [3,4]. Furthermore, some studies revealed that the bioactive potential of fish milt could be enhanced following a hydrolysis step [5,6]. In the case of herring milt hydrolysate (HMH), antioxidant and anti-inflammatory activities were reported in vitro [7,8] while the ability of this latter to modulate diabetes pathology was demonstrated at both in vitro and in vivo levels [2,7,9,10]. However, HMH, like other fish hydrolysates and fish-related products, has the main disadvantage to be associated with unpleasant odors and this limits its use in spite of the aforementioned promising aspects [11,12]. Therefore, there is a real need to address this major issue. 

Generally, off-flavors in marine products and related products including hydrolysates are due to the contribution of various compounds being all volatile, low-molecular-weight and hydrophobic [11]. In marine materials, trimethylamine (TMA) and dimethylamine (DMA), two amines originating from the degradation of trimethylamine oxide (TMAO), are the most well-known sources of unpleasant odors [13,14]. In addition to these nitrogenous compounds, other compounds belonging to various chemical groups such as aldehydes, ketones or sulfur-containing compounds have been reported to be an important cause of off-flavors as well [11]. In the case of HMH more particularly, the presence of TMA, DMA and TMAO has recently been evidenced in addition to the major contribution to its odor of 15 compounds belonging mainly to the aldehyde chemical group [12]. Interestingly, among these 15 compounds, most of them have already been identified as being important contributors to the odor of other fish products except for 1-methyl-1H-tetrazole and (Z)-6-octen-2-one that would be more characteristic of the HMH smell [12]. 

To remove these compounds responsible for off-flavors, several strategies have already been designed so far. Based on their mechanism of action, they can be classified into three categories: biological, chemical and physical strategies. Despite the fact that these strategies have already proven their relevance at different degrees, all seem to be associated with some disadvantages. Indeed, biological strategies implying the use of microorganisms have a limited action spectrum and their mechanisms remain unclear [11,15]. Chemical strategies include ozonation and the use of antioxidants. As ozonation is based on the use of a powerful oxidizing agent, ozone, side reactions risking to damage the product of interest may arise [16] whereas antioxidants are more used to prevent the formation of off-flavors rather than to remove them [11]. Physical strategies refer to extraction and adsorption processes. As extraction processes generally involve high temperatures, thermolabile compounds can be altered [17] whereas the efficiency of adsorption processes is limited [11]. Therefore, there is a real need to develop an effective deodorization method. Recently, Todeschini, et al. (2020) showed that a deaerator treatment conducted on HMH at neutral pH preliminary stirred overnight with nitrogen as well as a simple alkalization of this latter could decrease its most potent odor-active compound contents as well as those of TMAO [12]. Nevertheless, this study neither analyzed the respective role of pH, overnight stirring with nitrogen and deaerator treatment nor the effect of a combination of these three factors with regards to the observed decrease in the targeted odorous compounds. Since the two treatments introduced by Todeschini et al. (2020) present the advantages to be easy to operate without being time consuming and demanding in terms of materials compared to the deodorization strategies commonly used, they are worth to be further investigated. 

In this context, the objectives of the present research were (1) to deepen the knowledge regarding the impacts of pH, overnight stirring with nitrogen, deaerator treatment on TMAO, TMA and DMA as well as on the main contributors to the odor of HMH and (2) to identify the optimal conditions leading to the best removal rate of these odorous compounds.

## 2. Materials and Methods

### 2.1. Materials

#### 2.1.1. Chemicals

Methylene chloride and nonyl acetate were purchased from Fisher (Montréal, QC, Canada). Hydrochloric acid (HCl) and sodium hydroxide (NaOH) were obtained from VWR (Montréal, QC, Canada). TMAO, TMA and DMA standards were purchased from Sigma-Aldrich (St-Louis, MO, USA). Helium and nitrogen were from Praxair (Mississauga, ON, Canada). Oxygerm, Blizzard and Extrem solutions were obtained from Sani Marc (Victoriaville, QC, Canada).

#### 2.1.2. Herring Milt Hydrolysate (HMH)

HMH powder was supplied by Ocean NutraSciences (Matane, QC, Canada) and was stored under vacuum and protected from light at −30 °C before its use. The chemical composition of HMH dry powder was the following: 79.27 ± 0.17% of total nitrogen, 48.28 ± 0.44% of peptides, 27.30 ± 3.57% of nucleic acids, 18.48 ± 1.27% of lipids and 11.55 ± 0.20% of ashes [7]. 

### 2.2. Methods

#### 2.2.1. Protocol

**Figure 1:**
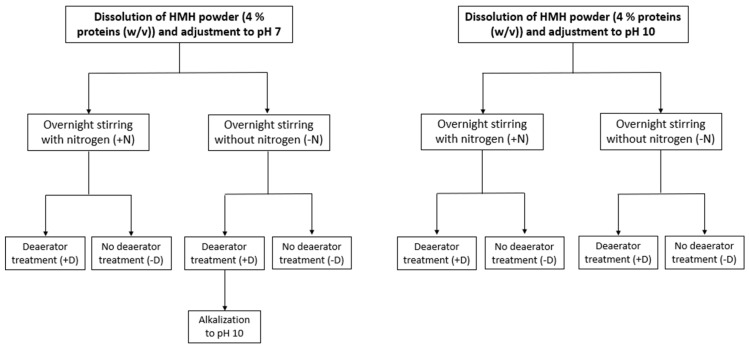
Summary of the eleven treatments performed on herring milt hydrolysate (HMH).

Experiments were conducted on HMH solutions dissolved to 4% proteins (*w*/*v*) and whose initial pH of 7.3 was adjusted either to pH 7 with 6 M HCl or to pH 10 with 6 M NaOH. HMH solutions were then stirred overnight at 4 °C, while protected from light by being covered with aluminum foil, either with nitrogen or without depending on the tested conditions before being finally treated or not by a deaerator device model ERV2 (Koruma, Neuenburg, Germany) (Figure 1). Treatment by deaerator was conducted for 30 min under 100 Torr in a closed circuit in accordance with the procedure described by Todeschini et al. (2020) [12]. Before use, the deaerator was decontaminated with Oxygerm solution consisting in a mix of organic acids and powerful oxidizers. Following each assay, the deaerator underwent another cleaning step with Blizzard and Extrem solutions consisting, respectively, in a foaming chlorinated alkaline degreaser and a mix of caustic soda, sequestering and wetting agents. An additional alkalization step to pH 10 with 6 M NaOH was carried out on the HMH solution at pH 7 stirred overnight without nitrogen and treated by deaerator (Figure 1). This was done to assess if a final alkalization step could enhance the ability of the deaerator to decrease the volatile compound content of HMH solution treated at pH 7. Samples were collected just after dissolution of HMH powder, once pH was adjusted, and then at each step of the protocol. They were stored at −20 °C protected from light before being studied in terms of volatile compound contents. All the tested conditions led to a total of eleven different treatments, summarized in Figure 1, performed in three replicates and in random order. 

#### 2.2.2. Analyses

a. Volatile Compound Analyses

*DMA, TMA and TMAO contents*

To assess the DMA, TMA and TMAO contents, headspace (HS) gas chromatography (GC) with a nitrogen-phosphorus detector (NPD) was used. Samples were prepared as previously described by Todeschini et al. (2020) [12]. Summarily, 2 mL of standards or samples were transferred into vials of 20 mL and alkalized to pH 10 with 0.1 M or 5 M NaOH solutions depending on their initial pH to avoid any dilution effect. Samples were then equilibrated at 90 °C for 10 min in a 7697A headspace sampler (Agilent Technologies, Santa Clara, CA, USA). The combination of both alkalization and heating aimed to maximize the recovery of DMA, TMA and TMAO molecules. After that, 1 mL of headspace loop was injected into a 7890B GC (Agilent Technologies, Santa Clara, CA, USA) equipped with a NPD. The injection was done in split mode with a ratio of 1:5 and the injector temperature was set at 250 °C. The loop and transfer line were, respectively, at 110 °C and 115 °C. A CP-Volamine capillary column (30 m length × 0.32 mm id) (Agilent Technologies, Santa Clara, CA, USA) was used to separate the volatile amines. Helium was the carrier gas and circulated at a constant flow rate of 3 mL/min. The oven temperature was set up from 50 °C (temperature maintained for 4 min) to 200 °C at 25 °C/min. The detector temperature was at 300 °C. Calibration curves made of known concentrations of DMA, TMA and TMAO standards (from 2.5 ppm to 10 ppm for DMA and TMA; from 25 ppm to 755 ppm for TMAO) were used to determine their concentration in the different samples. Data were treated with Open Lab CDS software version A.03.02.023 (Agilent Technologies, Santa Clara, CA, USA). 

*Most Potent Odor-Active Compound Contents*

Most Potent Odor-Active Compound Extraction

Volatile compounds were extracted as previously mentioned by Tremblay et al. (2020) [18]. Cotton balls were used to absorb 45 mL of liquid samples. Then, they were enclosed in glass tubes in which air circulated for 1h to allow the recovery of volatile compounds on a divinylbenzene column (HayeSep^®^ Q 80/100, Bandera, TX, USA) at room temperature. Afterwards, volatile compounds were eluted with 150 µL of solvent consisting in dichloromethane and nonyl acetate used as an internal standard at a concentration of 3.71 × 10^−5^ M. Samples were stored at −80 °C before their analysis.

Most Potent Odor-Active Compound Determination

A gas chromatography-mass spectrometry (GC-MS) system composed of a 7890B GC (Agilent Technologies, Santa Clara, CA, USA) involving a 5977B mass selective detector (MSD) and a high efficiency source (Agilent Technologies, Santa Clara, CA, USA) was used. Extracts (1.8 µL) were analyzed similarly to Tremblay et al. (2020) [18]. They were injected into a DB-5MS Ultra Inert capillary column (30 m length × 250 µm id, 1 µm thickness) (Agilent Technologies, Santa Clara, CA, USA) in splitless mode. Helium was the carrier gas and circulated at a constant flow rate of 1.3 mL/min. The oven temperature was set up from 35 °C (temperature maintained for 1.46 min) to 47 °C at 6 °C/min and to 250 °C (temperature maintained for 3 min) at 10 °C/min. The MSD source and MSD quad were, respectively, at 230 °C and 150 °C. Mass spectra were analyzed at an ionization energy of 70 eV. MSD ran in scan mode over a m/z range between 30 and 250 atomic mass unit (a.m.u) with a scan rate of 6.1 scans/s. Volatile compounds were identified by comparing their mass spectra with those of the NIST 17.L mass spectral database (National Institute of Standards and Technology, Gaithersburg, MD, USA). Only the compounds considered as the most potent odor-active ones of HMH [12] were studied and the area under their peak was used to assess their abundance in the different samples. Data were treated with MassHunter Qualitative Analysis software version B.07.00 (Agilent Technologies, Santa Clara, CA, USA).

b. Sensory Analysis

Among all the tested conditions, five were subjected to a sensory analysis. These five conditions were selected based on HS-GC-NPD and GC-MS results and corresponded to the two conditions leading to the best removal of the targeted compounds, to the two worst and to an intermediate one. This was done to assess if differences identified by analytical tools could be confirmed by human perception. Sensory analysis procedure was adapted from Chen et al. (2016) [11]. A panel of twenty judges, made of fifteen females and five males between 23 and 51 years old, joined the sensory analysis. Panel members were selected based on their motivation and availability without having followed a preliminary training session to be more representative of ordinary consumers. Samples were assessed at room temperature, right after leaving a 4 °C cold chamber, in crucibles of 40 mL capacity containing 5 mL of HMH solution and covered by plastic Petri dishes to avoid any loss of volatile compounds. Samples were ascribed a three-digit code and were placed in random order. The sensory assessment consisted in ranking the five selected conditions according to their odor intensity from the least to the most odorous. To do this, panel members were asked to gently remove plastic Petri dishes to sniff the headspace above the samples by normal breaths and to replace the cover quickly. A score (S) was given for each assessed condition where S was obtained according to Equation (1) [11].

(1) $$S=\sum^{\;}R_{i}$$

*R_i_* refers to the rank ascribed by each panel member for a given sample. Therefore, the condition leading to the lowest value of *S* was those considered as being the least odorous while those leading to the highest value of *S* was those considered as the most odorous.

c. Statistical Analyses

Statistical analyses were done using SAS software version 9.4 for windows (SAS Institute Inc., Cary, NC, USA). Analyses of variance (ANOVA) were carried out on HS-GC-NPD and GC-MS data and Tukey test (α = 0.05 as probability level) was used for the comparison of the different treatments. Regarding sensory analysis results, the non-parametric Friedman test was first used to detect any difference among all the selected treatments and then, Wilcoxon test (α = 0.05 as probability level) was conducted to compare the treatments two by two to identify those being different from the other ones.

## 3. Results and Discussion

### 3.1. Volatile Compound Analyses

#### 3.1.1. DMA, TMA and TMAO Contents

The content in DMA, TMA and TMAO of HMH solutions after dissolution and HMH solutions produced over the different tested conditions is shown in Table 1. First, regarding the HMH solutions after dissolution at pH 7 and 10, higher DMA, TMA and TMAO contents were detected for the solution at pH 10 than for those at pH 7 (*p* < 0.05). This was consistent with the ability of basic pH to increase the free contents of these molecules resulting in their better recovery [19,20]. Indeed, HMH, similarly to other complex matrices, involves a blend of various compounds including amino acids, peptides, nucleic acids and all of them can interact with DMA, TMA and TMAO. For this reason, to be detected, DMA, TMA and TMAO have to be present under a free form meaning that they should not be involved in interactions and basic conditions are well reported for their propensity to release DMA, TMA and TMAO from these potential interactions [19,20]. Therefore, the lower content in DMA, TMA and TMAO detected for the pH 7 solution after dissolution could be, in fact, related to their potential involvement in interactions with other constituents of HMH. Indeed, based on the pKa values of DMA and TMA, respectively equal to 10.70 and 9.80 [21], these two compounds were mainly present under their acid form at pH 7. This means that both DMA and TMA were positively charged at this pH value making them more likely to establish electrostatic interactions with other negatively charged constituents of HMH hampering, thus, their release and resulting in their lower detection at pH 7. These negatively charged constituents could be the C-term of peptides (pKa ~ 2.1) in addition to the side-chains of free or bound glutamic and aspartic acids (pKa ~ 4.0) [22]. Besides, HMH contains nucleic acids. Since these compounds are globally negatively charged, they could, as well, establish electrostatic interactions with both DMA and TMA. Regarding TMAO more specifically, this compound presents the specificity to carry a negative charge and a positive one at pH 7 making it likely, as well, to establish electrostatic interactions with other constituents of HMH. Similarly to what was mentioned for DMA and TMA, the positive charge of TMAO could interact with the negatively charged species of HMH such as the C-term of peptides (pKa ~ 2.1) or the side-chains of free or bound glutamic and aspartic acids (pKa ~ 4.0) at pH 7 [22] as well as nucleic acids. On the contrary, the negative charge of TMAO could interact with the positively charged species such as the N-Term of peptides (pKa ~ 9.8) or the side-chains of lysine (pKa ~ 10.5) and arginine (pKa ~ 12.5)) in both their bound and free forms [22]. Interestingly, HMH involves a high content in arginine, mainly present under a free form, and this would give credit to the latter explanation [7]. At pH 10, a certain part of these interactions would have been broken especially for TMA being neutral under this pH value, explaining thus the higher detected content. Besides, no difference was observed between the HMH solutions at pH 7 stirred overnight without nitrogen (pH 7 -N -D) or with nitrogen (pH 7 +N -D) and those after dissolution (*p* > 0.05). On the one hand, this suggests that a simple stirring, independently of the presence of nitrogen, was not sufficient to increase the release of the targeted compounds by breaking, in particular, the potential interactions taking place between them and the other constituents of HMH. It is noteworthy to mention that the fact that the stirring step, regardless of the use of nitrogen, did not bring about a better detection of the targeted compounds, in this study, was not in line with the stir bar sorptive extraction method that uses stir bars to improve the release of volatile compounds [23]. As temperature is a limiting factor with regards to this procedure [24], the fact that the overnight stirring of HMH solutions was conducted at 4 °C and not at higher temperatures, to prevent the polyunsaturated fatty acids of HMH from degradation in addition to microbiological concerns, could explain this discrepancy. On the other hand, the fact that no difference was observed between HMH solution after dissolution and the pH 7 -N -D or pH 7 +N -D HMH ones might indicate as well that DMA, TMA and TMAO were distributed homogeneously in solution directly after dissolution. This would be corroborated by the general low standard deviation values with regards to their corresponding means (Table 1). Moreover, the fact that no significant difference was observed between the pH 7 -N -D and pH 7 +N -D hydrolysate solutions showed that at this pH value, nitrogen had no effect on the targeted compounds. This could be consistent with the fact that nitrogen flushing is, in general, used to prevent the degradation of food products by limiting, in particular the oxidation of lipids, and not to modify their current composition including those in volatile compounds [25]. Concerning the effect of the overnight stirring on HMH solutions at pH 10, no change was observed between the solutions stirred without (pH 10 -N -D) or with nitrogen (pH 10 +N -D) and those after dissolution (*p* > 0.05) except for the TMA content that was lower for the pH 10 +N -D condition (*p* < 0.05). Since the release of TMA was increased under basic conditions [19,20], a punctual loss of this compound, promoted by the presence of nitrogen, would have occurred during the overnight stirring explaining such a result. Interestingly, the pH effect previously observed between the HMH solutions at pH 7 and 10 after dissolution was still visible for DMA and TMA after the overnight stirring, regardless of the presence of nitrogen, but not anymore for TMAO. As the pH effect was less pronounced for TMAO than for DMA and TMA between the solutions at pH 7 and 10 after dissolution, this might explain the reason why this pH trend was not evidenced for TMAO afterwards. Therefore, based on the results obtained for the solutions at pH 7 and 10 just after dissolution and after the overnight stirring with or without nitrogen, it appeared that neither the stirring step nor the presence of nitrogen impacted the content in DMA, TMA and TMAO. This means that in case of further treatment, this latter could be performed directly on a solution just after dissolution. This would save time as well as materials. Concerning the effect of a deaerator, no change was observed for the pH 7 -N +D and pH 7 +N +D hydrolysate solutions compared to those stirred in the same conditions but not treated by deaerator (*p* > 0.05). However, a decrease in the TMA content was noted for the pH 10 -N +D and pH 10 +N +D HMH solutions compared to those stirred in the same conditions but not treated by deaerator (*p* < 0.05). This represents a decrease in the TMA content of 80% in comparison with the HMH solution at pH 10 just after dissolution. This huge effect of the combination of both pH and deaerator treatment on the TMA content was confirmed by statistical analyses (*p* < 0.05). Besides, regarding the DMA content of the pH 10 -N +D and pH 10 +N +D HMH solutions, even if it was not perceived in terms of statistical differences (*p* > 0.05), a downward trend could be noted, while no change was evidenced for TMAO in these conditions (*p* > 0.05). Since deaerator is a device used to remove gas from a matrix, volatile compounds due to their volatile state, are removed at the same time. The fact that the deaerator was more effective to decrease the DMA and TMA contents at pH 10 than at pH 7 might be explained by its better ability to remove these compounds resulting from their higher release under alkaline conditions [19,20]. Surprisingly, the deaerator was more effective to remove TMA than DMA even though these two compounds have a similar structure and would have been expected to evidence the same behavior. Nevertheless, based on the pKa values of TMA and DMA, respectively equal to 9.80 and 10.70 [21], TMA was mainly present under its basic form while DMA was mainly present under its acid one at pH 10. This means that TMA was under a neutral form while DMA was under a positively charged one. Therefore, DMA could be still involved in electrostatic interactions with the negatively charged constituents of HMH hampering, thus, the deaerator to remove it as easily as TMA at pH 10. Therefore, performing the deaerator treatment at higher pH value, such as pH 11, would enhance the removal of DMA as this compound would not be charged anymore. Moreover, based on what was mentioned previously, the ineffectiveness of the deaerator treatment to remove both TMA and DMA at pH 7, would be linked to their propensity to electrostatically interact with other components of HMH. This explanation could be involved as well for TMAO at both pH values. Indeed, since TMAO carries a negative charge and a positive one at pH 7 and 10, this compound was able to electrostatically interact with other charged constituents of HMH decreasing, thus, the ability of deaerator to remove it. Another interesting point to note is that the DMA, TMA and TMAO contents detected for the pH 7 -N +D and pH 7 +N +D solutions, on the one hand, and for the pH 10 -N +D and pH 10 +N +D solutions, on the other hand, were similar for the same value of pH (*p* > 0.05). This means that there was no effect of the stirring conditions on the deaerator treatment and this was confirmed by statistical analyses (*p* > 0.05). Finally, regarding the impact of a further alkalization step conducted on the pH 7 -N +D HMH solution, the detected content in DMA and TMA was higher than those in the pH 7 -N +D one (*p* < 0.05) while the detected content in TMAO was similar in both (*p* > 0.05). The increase in the detected TMA and DMA contents following the alkalization step was in line with what was observed so far regarding the ability of basic conditions to improve the release of these compounds [19,20]. Nonetheless, in a deodorization context, as the final objective is to reduce the content in odorous compounds, conducting an additional alkalization step appeared to be irrelevant as it brought about a higher content in free DMA and TMA. Thus, based on DMA, TMA and TMAO results, the treatments allowing the best removal rate of these compounds, were the pH 10 -N +D and pH 10 +N +D ones. Furthermore, these treatments appeared to be more effective in terms of TMA removal, more specifically, reaching a removal rate of 80%, in comparison with other deodorization strategies described in the literature. Indeed, Park et al. (2020) reported a decrease in the TMA content from 45 to 62% in spoiled fish by lactic acid bacteria [26] while Chen et al. (2016) observed no effect of a yeast extract on the TMA content of a clam hydrolysate [11]. Using adsorption strategies, a TMA removal rate of 73.3% was achieved by Boraphech and Thiravetyan (2015) on activated carbon [27] whereas Chen et al. (2016) found the adsorption on activated carbon ineffective to reduce the TMA content of a clam hydrolysate [11]. Interestingly, Chung and Lee (2009) demonstrated slightly higher TMA removal rates using adsorption on zeolites with regards to the present study. Since they used a pure solution of TMA and not a complex matrix made of various compounds such as HMH, this may explain such results [28]. Therefore, comparisons with data obtained from the literature might indicate the high potential of the treatments developed in the present work to be used as a new avenue to deodorize complex matrices such as HMH. It is worth mentioning that the comparison between the present deodorization strategies with other ones in terms of DMA and TMAO removal efficiency was not possible since the literature mainly reported studies dealing with TMA removal.

#### 3.1.2. Most Potent Odor-Active Compound Contents

The abundance of the most potent odor-active compounds of HMH solutions after dissolution and of HMH solutions produced over the different tested conditions is presented in Table 2. First, no significant difference was globally noticed between HMH just after dissolution at pH 7 and HMH stirred overnight with (pH 7 +N -D) or without nitrogen (pH 7 -N -D) (*p* > 0.05). Similarly to what was mentioned for DMA, TMA and TMAO, this means that a simple stirring, independently of the presence of nitrogen, was not sufficient enough to increase the release of the targeted compounds and that these compounds were distributed homogeneously in solution directly after dissolution as well. However, this was not totally confirmed for 3-methylbutanal, 2-methylbutanal, pentanal and 1-methyl-1H-tetrazole. Indeed, the 3-methylbutanal, 2-methylbutanal and pentanal contents were lower in the solutions stirred overnight, regardless of the presence of nitrogen, while 1-methyl-1H-tetrazole was not even detected after the stirring step (*p* < 0.05). Based on what was mentioned before, this might indicate that either these compounds, unlike the other ones, would have been lost due to their volatile state during the stirring step or that, on the contrary, the stirring step allowed these compounds to better interact with the other constituents of HMH hampering their optimal detection afterwards. At that stage, it was not obvious to clearly choose one explanation rather than the other one. Moreover, the fact that no significant difference was noticed between the pH 7 -N -D and pH 7 +N -D solutions showed that at this pH value, nitrogen had no effect on the most potent odor-active compounds of HMH and this was consistent with what was observed for DMA, TMA and TMAO. Then, with regards to the impact of a deaerator treatment conducted on a HMH solution that was stirred overnight without nitrogen at pH 7 (pH 7 -N +D), no significant change in the content in the most potent odor-active compounds was observed compared to the pH 7 -N -D HMH solution (*p* > 0.05) except for (Z)-6-octen-2-one whose abundance dropped by 22.5% (*p* < 0.05). It is noteworthy to mention that the abundance of nine other compounds namely, 3-methylbutanal, 2-methylbutanal, 2,3-pentanedione, pentanal, (Z)-4-heptenal, methional, benzaldehyde, octanal, 2-nonanone would decrease for the pH 7 -N +D HMH solution in comparison with the pH 7 -N -D one even if it was not perceived in terms of statistical differences. Besides, compared to the HMH solution at pH 7 just after dissolution, the abundance of the five following compounds dropped by at least 35% for the pH 7 -N +D HMH solution (*p* < 0.05): 3-methylbutanal, 2-methylbutanal, pentanal, (Z)-4-heptenal and 2-nonanone. Concerning the effect of a deaerator treatment on the HMH solution stirred overnight with nitrogen (pH 7 +N +D), no significant difference was observed compared to the pH 7 +N -D HMH one (*p* > 0.05). Nevertheless, similarly to what was mentioned before, even if it was not perceived in terms of statistical differences, the abundance of 3-methylbutanal, 2-methylbutanal, hexanal, (Z)-4-heptenal, heptanal, benzaldehyde, (Z)-6-octen-2-one, (E,E)-2,4-heptadienal, octanal and 2-nonanone would decrease after the deaerator treatment as well. Moreover, compared to the HMH solution just after dissolution, the abundance of 3-methylbutanal, 2-methylbutanal, pentanal, (Z)-4-heptenal and 2-nonanone dropped by at least 55% for the pH 7 +N +D HMH solution (*p* < 0.05). As the aim of a deaerator is to remove gas from a matrix, this device also takes part in the elimination of HMH odorous compounds due to their volatile state and this trend was confirmed for the most potent odor-active compounds of HMH as well as for TMA and DMA before. Furthermore, it is interesting to note that the pH 7 -N +D and pH 7 +N +D HMH solutions had a similar content (*p* > 0.05). This indicated that, similarly to DMA, TMA and TMAO, there was no synergistic effect between the overnight stirring, with or without nitrogen, and the deaerator treatment for the HMH solutions at pH 7 and this was confirmed by statistical analyses (*p* > 0.05). In addition to the aforementioned treatments, another one consisting in an alkalization step to pH 10 of the pH 7 -N +D HMH solution was conducted. This treatment resulted in a decrease in hexanal, (E,Z)-2,6-nonadienal (*p* < 0.05) and in the total removal of both (E,E)-2,4-heptadienal and octanal compared to the pH 7 -N +D HMH solution. Interestingly, compared to the HMH solution at pH 7 just after dissolution, the alkalized pH 7 -N +D HMH solution allowed a decrease from around 50 to 100% of these thirteen compounds: 3-methylbutanal, 2-methylbutanal, 1-methyl-1H-tetrazole, 2,3-pentanedione, pentanal, hexanal, (Z)-4-heptenal, methional, benzaldehyde, (E,E)-2,4-heptadienal, octanal, 2-nonanone and (E,Z)-2,6-nonadienal (*p* < 0.05). Interestingly, the decreases in hexanal, (E,E)-2,4-heptadienal, octanal and (E,Z)-2,6-nonadienal were observed after the alkalization step of the pH 7 -N +D HMH solution and not directly on the pH 7 -N +D one after the deaerator treatment in comparison with the hydrolysate solution just after dissolution. This would mean that these four compounds would be more impacted by the addition of NaOH while the other ones would be more affected by the deaerator treatment. Therefore, it seems that the main removal mechanism of 3-methylbutanal, 2-methylbutanal, pentanal, (Z)-4-heptenal, benzaldehyde and 2-nonanone would be simply linked to their volatile state at pH 7. Statistical analyses confirmed the high impact of deaerator treatment on 2-methylbutanal, pentanal and benzaldehyde (*p* < 0.05). Regarding the impact of NaOH addition, Todeschini et al. (2020) already observed a drop in the abundance of the most potent odor-active compounds of HMH while pH rose from 7 to 10 and formulated three hypotheses to explain such a decrease. The first one was that at pH 10, a certain part of volatile compounds would establish covalent bonds with the other constituents of HMH. The second one involved a potential alteration of these compounds due to the addition of NaOH resulting in hindering their detection. Finally, the last one dealt with their possible loss due to their volatile state following their promoted release under basic conditions similarly to DMA, TMA and TMAO [12]. Regarding the first hypothesis, as samples were collected for analyses just after the alkalization step of the pH 7 -N +D HMH solution, this should be too short in time to allow the establishment of covalent bonds between hexanal, octanal, (E,E)-2,4-heptadienal and (E,Z)-2,6-nonadienal and the other constituents of HMH. This was corroborated by the study of Zhou et al. (1999) in which they observed the development of covalent bindings between unsaturated aldehydes and amino acids materials after one hour of incubation [29]. Furthermore, among aldehyde compounds, those that are the most described to be involved in covalent bonds are monounsaturated ones [30]. Indeed, the presence of a carbonyl group C=O, in addition to the double covalent bonds between two carbon atoms C=C, would make them more reactive than saturated aldehydes, such as hexanal and octanal, especially in the presence of nucleophilic molecules [30,31]. With regards to (E,E)-2,4-heptadienal and (E,Z)-2,6-nonadienal, their propensity to establish covalent bonds with other nonvolatile molecules was less described in the literature. The fact that (E,E)-2,4-heptadienal and (E,Z)-2,6-nonadienal are polyunsaturated aldehydes may lead to stearic effect decreasing their potential to easily react with nucleophilic compounds on the contrary to monounsaturated ones. Thus, this would indicate that this first hypothesis could not be involved in the present case. To totally exclude this hypothesis, it could be relevant to analyze the amino acid content of HMH. Indeed, since amino-acid-containing materials are the main constituents establishing covalent bonds with aldehyde compounds resulting in the formation of Schiff bases and Michael adducts [29,31], an absence of change regarding the amino acid content of HMH at pH 10 would confirm that this hypothesis is not appropriate. Then, regarding the second hypothesis involving an alteration of volatile compounds by NaOH, it seems that this explanation would not be consistent in that case due to the too short time lapse between the alkalization of the HMH solution and the sampling as well. Therefore, the most plausible explanation would be linked to the third hypothesis, at that stage, and would be that the decreases in hexanal, octanal, (E,E)-2,4-heptadinal and (E,Z)-2,6-nonadienal imply their simple loss following the addition of NaOH. Nonetheless, based on the results obtained for DMA, TMA and TMAO, an increase in their contents was observed at pH 10 following their better release under basic conditions and thus, their better recovery. Thus, this might rather indicate that nitrogenous and aldehyde compounds would evidence opposite behavior as a function of pH value and that it would not be appropriate to extrapolate the properties of nitrogenous compounds to aldehyde ones leading to reject this third hypothesis as well. In fact, while numerous studies reported the impact of basic pH on the volatility of nitrogenous compounds, no data are available regarding those of aldehydes except for the study of Shimoda et al. (1996), in which they reported a decrease in the concentration of aldehydes of fish sauce under alkaline conditions in comparison with acid ones [32]. Therefore, this led to formulate a new hypothesis regarding the observed decrease in the content in aldehydes. This new hypothesis would be that, on the contrary to nitrogenous compounds, aldehyde ones would evidence a drop in their volatile state hampering their optimal release and thus bringing about their underestimated detection at basic pH whereas acid conditions would promote their release. Thus, the lower content in hexanal, octanal, (E,E,)-2,4-heptadienal and (E,Z)-2,6-nonadienal observed for the pH 7 -N +D + alkalization pH 10 condition would be related, in fact, to a decrease in their volatile state. It appeared from the results that the most potent odor-active compounds of HMH at pH 7 were in general more impacted by the deaerator treatment independently of the condition of stirring. Thus, the deaerator treatment could be performed just right after dissolution of HMH powder, without an overnight stirring and without the use of nitrogen, and this would allow to save time as well as materials.

Regarding HMH at pH 10, no change was globally perceived between the HMH solution at pH 10 after dissolution and after the overnight stirring with (pH 10 +N -D) or without nitrogen (pH 10 -N -D) (*p* > 0.05) except for hexanal, (Z)-4-heptenal and octanal whose content tended to decrease for the pH 10 -N -D condition. Similarly to what was mentioned for HMH at pH 7, this would imply that independently of the presence of nitrogen, an overnight stirring was not sufficient enough to increase the release of most of the targeted compounds and that most of them were distributed rapidly in solution after dissolution as well. On the contrary, regarding hexanal, (Z)-4-heptenal and octanal, the observed decrease might indicate that the stirring step in absence of nitrogen either led to a loss of these compounds probably related to their volatile state or promoted their interactions with other constituents of HMH resulting in their lower detection. In addition, the global absence of difference between the HMH solutions stirred with and without nitrogen would indicate that at pH 10, nitrogen had no impact on the abundance of the targeted compounds similarly to what was observed at pH 7. Interestingly, the comparison between pH 7 and pH 10 conditions showed that the abundance of 3-methylbutanal, 2-methylbutanal, pentanal, hexanal, (Z)-4-heptenal and octanal varied as a function of both pH and overnight stirring independently of the presence of nitrogen (*p* < 0.05). Nevertheless, depending on the aldehyde compound, the combination of these two factors did not evidence the same impact. Indeed, the content in 3-methylbutanal, 2-methylbutanal and pentanal tended to decrease during the overnight stirring at pH 7 but not at pH 10. On the contrary, the content in hexanal, (Z)-4-heptenal and octanal tended to decrease during the overnight stirring at pH 10 but not at pH 7. This might indicate that the impact of the combination of pH and stirring conditions on aldehyde compounds would depend mainly on their chain length. These results seem to give credit to the new hypothesis formulated previously indicating that aldehydes would evidence a drop in their volatile state under alkaline conditions hampering their optimal release whereas acid conditions would enhance their release. Based on this, it looks like that the potential decrease in the volatility of aldehydes involving six carbon atoms or more in their chain length under basic pH would promote their propensity to interact with the components of HMH. This trend would not be as obvious to be observed for 3-methylbutanal, 2-methylbutanal and pentanal since these aldehydes involve a smaller chain length and therefore, present by their nature, a higher volatility regardless of the pH conditions. On the contrary, it seems that the potential increase in volatility of aldehydes under acid conditions, would be perceived even at neutral pH for compounds involving five carbon in their chain length or less, as at pH 7, a decrease in the abundance of 3-methylbutanal, 2-methylbutanal and pentanal was observed. To confirm this new hypothesis, it could be relevant to conduct a further acidification step on an alkalized solution to verify the impact on the concentration of aldehydes. Surprisingly, the abundance of heptanal, a saturated aldehyde with structure similar to hexanal and octanal, was not shown to vary as a function of both pH and stirring (*p* > 0.05). The fact that the standard deviation, for the condition pH 7 -N -D, was quite high compared to the corresponding abundance value of heptanal might have introduced a bias in the results explaining that such a trend was not observed in that case. Then, regarding the effect of a deaerator treatment on the HMH solution stirred overnight without nitrogen (pH 10 -N +D), only the abundance of (Z)-6-octen-2-one and 2-nonanone dropped significantly compared to the pH 10 -N -D HMH solution (*p* < 0.05). In addition, even if it was not perceived in terms of statistical differences, the abundance of 3-methylbutanal, 2-methylbutanal, 2,3-pentanedione, pentanal, hexanal and benzaldehyde would decrease as well between the pH 10 -N -D and pH 10 -N +D HMH solutions. Compared to HMH solution at pH 10 just after dissolution, no change in the targeted compounds content was observed (*p* > 0.05) except for (Z)-6-octen-2-one and 2-nonanone whose abundance dropped by 68 and 80% respectively in pH 10 -N +D HMH (*p* < 0.05). Concerning the impact of a deaerator treatment on HMH solution at pH 10 stirred overnight with nitrogen (pH 10 +N +D), only a decrease in the content in (Z)-6-octen-2-one and 2-nonanone was observed compared to the pH 10 +N -D HMH solution (*p* < 0.05). Besides, a downward trend could be observed as well for 3-methylbutanal, pentanal, (Z)-4-heptenal and benzaldehyde even if it was not confirmed by statistical analyses. Compared to HMH solution at pH 10 just after dissolution, no change was observed (*p* > 0.05) for the pH 10 +N +D HMH solution except for (Z)-6-octen-2-one and 2-nonanone whose abundance dropped by 55 and 70% respectively. As mentioned previously, as deaerator aims to remove gas, volatile compounds are removed at the same time and this was globally confirmed for the most potent odor-active compounds of HMH at pH 10 as well. Besides, no difference was observed between the pH 10 -N +D and pH 10 +N +D HMH solutions (*p* > 0.05). Similarly to what was mentioned for the pH 7 -N +D and pH 7 +N +D solutions, this was representative of an absence of synergy between the overnight stirring, with or without nitrogen, and the deaerator treatment for the HMH solutions at pH 10 and this was corroborated by statistical results (*p* > 0.05). It appeared from these results that the contents in (Z)-6-octen-2-one and 2-nonanone of the studied hydrolysate were the most impacted by a deaerator treatment at pH 10. However, this trend was not observed for 2,3-pentanedione, another targeted ketone compound, whose abundance mainly varied according to the pH value resulting in a lower content at pH 10 than at pH 7, independently of the tested conditions. Interestingly, regarding the solutions treated by deaerator at pH 7 and 10, it appeared, at first glance, that this treatment would be more effective to reduce the content in certain aldehydes such as 3-methylbutanal, 2-methylbutanal, pentanal or benzaldehyde at neutral pH as the final abundance of these compounds were lower at pH 7 than at pH 10. Nevertheless, statistical results revealed no significant effect of the combination of both pH and deaerator (*p* > 0.05). This even more corroborated the assumption that aldehydes would be better released at acid pH. For short-chain aldehydes such as 3-methylbutanal, 2-methylbutanal, pentanal, this would be already visible at neutral pH allowing a better removal rate of the deaerator treatment. Despite the fact that the deaerator treatment was effective to decrease the content in most of the targeted compounds, regardless of the pH value, for other compounds such as methional, (E,E)-2,4-heptadienal and (E,Z)-2,6-nonadienal, no effect was observed (*p* > 0.05). In fact, concerning these three compounds, only pH value had a great importance on their abundance (*p* < 0.05) leading to a lower content at pH 10 than at pH 7. On the one hand, the impact of basic conditions on (E,E)-2,4-heptadienal and (E,Z)-2,6-nonadienal was already observed for the pH 7 -N +D solution that underwent a further alkalization step. The fact that for all the treatments studied at pH 10, none of them had an impact on the abundance of (E,E)-2,4-heptadienal and (E,Z)-2,6-nonadienal would give credit to the hypothesis involving a decrease in their volatile state due to basic conditions explaining, thus, the inefficiency of these treatments. At that stage, it is worth to note as well, that the content in most of the targeted aldehydes was globally lower for the pH 7 -N +D + alkalization pH 10 condition than for the other conditions at pH 10. This corroborates the hypothesis involving an increase in the volatile state of aldehydes at acid pH and a decrease at basic pH. Indeed, based on the results obtained for the pH 7 -N +D + alkalization pH 10 condition, it appeared that performing a deaerator treatment on a HMH solution at neutral pH would lead to a high performance of the deaerator device to remove aldehydes based on the increase in their volatility and conducting a further alkalization step would bring about an additional decrease based, in that case, on a decrease in their volatile state resulting in their lower detection. On the other hand, regarding methional, none of the treatments conducted on HMH at pH 10 had an impact on its content and its abundance remained steady for all of them. Besides, as mentioned before, the content in methional was globally lower at pH 10 than at pH 7. Nevertheless, it was supposed previously that basic conditions would decrease the volatility of aldehyde compounds, but this would be less perceived for short-chain compounds involving five atoms of carbon in their chain or less. Methional is an aldehyde with four atoms of carbon in its chain but it presents, as well, the particularity to be a sulfur-containing compound. This difference in terms of structure could explain the fact that methional did not experiment the same behavior as other aldehydes with similar chain length. Finally, none of the conditions at pH 10 evidenced the presence of 1-methyl-1H-tetrazole even after the dissolution of HMH powder on the contrary to what was observed at pH 7. Since this compound is a nitrogenous one and since this chemical group is known to experiment an increase in volatility under basic conditions, this compound was probably lost very quickly at pH 10 explaining its was not detected afterwards. 

Globally, it appeared from these results that the overnight stirring step, regardless of the use of nitrogen, had globally no impact on the most potent odor-active compounds of HMH at both tested pH values. Therefore, in case of further treatment, it would be possible to conduct it on a solution directly after dissolution. It appears as well from the results that the deaerator was an effective mean to reduce the content in the targeted compounds. In a deodorization context, the treatments allowing the best removal of these compounds were the pH 7 -N +D, pH 7 +N +D and pH 7 -N +D + alkalization pH 10 ones. Interestingly, while comparing these treatments with other deodorization strategies targeting similar compounds, those identified in this study presented a higher efficiency. For instance, the pH 7 -N +D + alkalization pH 10 condition led to a decrease in hexanal of 75% in comparison with the pH 10 after dissolution condition whereas Pan et al. (2018) reported a decrease in this compound of 21% using activated carbon to deodorize the skin gelatin of tiger puffer fish [33]. Using another adsorption strategy based on the use of zeolites, Song et al. (2018) managed to remove 35% of the hexanal content of fish oil originating from tuna and anchovies by-products [34]. Güner et al. (2019) used adsorption on zeolites to remove (E,E)-2,4-heptadienal from fish oil and reached a removal rate of 60% [35] while in the present case, a total removal of this compound was obtained with the application of the pH 7 -N +D + alkalization pH 10 condition. The pH 7 +N +D condition brought about a decrease in 3-methylbutanal and 2-methylbutanal of respectively 60 and 85% in comparison with the pH 7 after dissolution condition. Shimoda et al. (2000) only reached a removal level of 3-methylbutanal of 45% in fish sauce with an extraction process based on the use of supercritical carbon dioxide [36] whereas the use of the bacterium *Staphylococcus xylosus* had no effect on the 2-methylbutanal content of fish sauce [15]. Similarly to what was mentioned previously for TMA, this seems to indicate the high performance of these new deodorization strategies to be applied to complex matrices. 

### 3.2. Sensory Analysis

To assess if differences observed by the HS-GC-NPD and GC-MS analyses were perceived by humans, sensory experiments were conducted. Among the eleven tested conditions, the five ones considered for this purpose were the following: pH 7 after dissolution, pH 10 after dissolution, pH 7 -N +D + alkalization pH 10, pH 7 +N +D and pH 10 -N +D conditions. The odorous S scores given by the panel members for each condition are presented in Table 3. Results revealed that panel members recognized the pH 7 and pH 10 after dissolution conditions as being the most odorous ones in comparison with the other tested conditions (*p* < 0.05). This was consistent with both HS-GC-NPD and GC-MS results. Indeed, the pH 7 after dissolution condition depicted the highest content in the most potent odor-active compounds of HMH while the pH 10 after dissolution one showed the highest content in TMA and DMA, two nitrogenous compounds well-known for their contribution to the off-flavors of marine products [13,19]. Then, the pH 10 -N +D and pH 7 -N +D + alkalization pH 10 conditions were identified as being the least odorous ones compared to the other selected conditions (*p* < 0.05). On the one hand, with regards to the pH 10 -N +D condition, the fact that this latter was perceived as the least odorous was in line with its lowest content in both TMA and DMA since, as mentioned before, these compounds are characterized by an unappealing smell that can be easily detected [13,19]. In addition, this condition showed, as well, a lower content in both (E,E)-2,4-heptadienal and (E,Z)-2,6-nonadienal in comparison with the conditions at pH 7. Interestingly, while these two compounds are present together, (E,Z)-2,6-nonadienal, initially characterized by a cucumber-like smell [37], boosts the off-flavor of (E,E)-2,4-heptadienal [38,39]. Thus, the lower abundance of these two alkadienals for the pH 10 -N +D might also explain its lowest S score. It is important to note that other synergistic effects may happen between the different targeted compounds but only few studies dealing with this problematic were reported in the literature. On the other hand, the low value of S score given by the panel to the pH 7 -N +D + alkalization pH 10 condition could be related to its low content in (E,E)-2,4-heptadienal and (E,Z)-2,6-nonadienal as well. Moreover, on the contrary to the pH 10 -N +D condition, the pH 7 -N +D + alkalization pH 10 one, showed a high content in both TMA and DMA. Nonetheless, the fact that this latter condition showed, as well, an overall lower content in the most potent odor-active compounds of HMH due to the general efficiency of the deaerator treatment at pH 7 to remove them, may explain the reason why this condition was chosen as one of the least odorous by the panel members. At that stage, it is worth to note that (Z)-4-heptenal was one of the most potent odor-active compounds whose abundance was lower for the pH 7 -N +D + alkalization pH 10 condition. Interestingly, (Z)-4-heptenal was reported in the literature to have the capacity to increase the smell of other compounds rather than being perceptible on its own [37,39]. Thus, this might have an impact as well on the overall lower smell of the pH 7 -N +D + alkalization pH 10 condition. Finally, the pH 7 +N +D condition received an intermediate S score and this could be representative of its overall lower content in the most potent odor-active compounds of HMH, similarly to what was mentioned for the pH 7 -N +D + alkalization pH 10 condition. However, its higher content in (E,E)-2,4-heptadienal and (E,Z)-2,6-nonadienal could explain the reason why this condition was globally perceived as being more odorous than the pH 7 -N +D + alkalization pH 10 and pH 10 -N +D ones. Therefore, it appeared that sensory analysis results were very consistent with those obtained by analytical tools and confirmed the efficiency of the pH 7 -N +D + alkalization pH 10 and pH 10 -N +D conditions to deodorize HMH.

## 4. Conclusions

This research aimed to deepen the knowledge regarding the impacts of pH, overnight stirring with nitrogen and treatment by deaerator on DMA, TMA and TMAO as well as on the most potent odor-active compounds of HMH to develop a new effective and easy-to-use deodorization method. Results showed that pH had a significant impact on the abundance of these compounds. Nevertheless, depending on the chemical group involved, different trends could be observed. Indeed, the nitrogenous compounds, namely DMA, TMA and TMAO, showed higher detected concentrations at pH 10 than at pH 7 due to the effect of alkaline conditions to increase their free contents while the opposite trend was observed for the most potent odor-active compounds of HMH evidencing higher detected concentrations at pH 7. In addition, it appeared from the results that the overnight stirring with or without nitrogen had globally no impact on the targeted compounds compared to the hydrolysate solution just after dissolution at a given pH value. Therefore, in case of further treatment, this latter could be conducted on the solution after dissolution directly. Interestingly, for certain targeted aldehydes, namely 3-methylbutanal, 2-methylbutanal, pentanal, hexanal, (Z)-4-heptenal and octanal, their abundance varied as a function of both pH and overnight stirring. However, depending on the chain length of the aldehyde compound, different behaviors could be observed. In fact, regarding aldehydes involving five atoms of carbon in their chain length or less, the overnight stirring at pH 7 led to a loss of these compounds due to the increase in their volatile state under this pH condition. On the contrary, aldehydes involving six atoms of carbon or more in their chain length depicted a lower content following the overnight stirring at pH 10. In that case, the observed drop could be related to their propensity to better interact with the other components of HMH due to their lower volatility under alkaline conditions. Then, the deaerator treatment had a huge impact on the abundance of the targeted compounds. More precisely, the deaerator treatment was more effective at pH 10 to remove DMA and TMA due to their higher release under basic conditions while the opposite trend was observed for the most potent odor-active compounds of HMH whose removal was superior at pH 7 due to their higher volatility at this pH value. The treatments showing the lowest content in DMA, TMA and TMAO were the pH 10 -N +D and pH 10 +N +D ones while those evidencing the lowest content in the most potent odor-active compounds of HMH were the pH 7 -N +D, pH 7 +N +D and pH 7 -N +D + alkalization pH 10 ones. Sensory analysis, carried out to confirm the differences identified by analytical tools by human perception, reported the pH 10 -N +D and pH 7 -N +D + alkalization pH 10 conditions as the least odorous ones and thus confirmed the results obtained by HS-GC-NPD and GC-MS procedures. Therefore, it effectively appeared from this study that conducting a deaerator treatment either on a HMH solution preliminary alkalized or on a HMH one at neutral pH that underwent then an additional alkalization step could be an effective way to reduce the odorous content of this matrix. To deepen the knowledge regarding these promising results, it would be relevant to consider using KOH instead of NaOH since the dissociation of this latter results in the generation of Na^+^ species closely related to hypertension concerns. In addition, it would be interesting to further investigate the potential effects of the pH change resulting from the alkalization procedure especially regarding the bioactivities of HMH proven, so far, for pH closed to neutral one.

## Figures and Tables

**Table 1:** Dimethylamine (DMA), Trimethylamine (TMA) and Trimethylamine Oxide (TMAO) contents of Herring Milt Hydrolysate (HMH) after dissolution and after the different treatments (mean ± standard deviation).

	pH 7 after Dissolution	pH 7 -N -D	pH 7 -N +D	pH 7 +N -D	pH 7 +N +D	pH 7 -N +D + Alkalization pH 10	pH 10 after Dissolution	pH 10 -N -D	pH 10 -N +D	pH 10 +N -D	pH 10 +N +D
DMA (ppm)	3.09 ± 0.45 **^c^**	3.18 ± 0.60 **^c^**	3.83 ± 0.19 **^bc^**	3.00 ± 0.17 **^c^**	3.79 ± 0.62 **^bc^**	5.87 ± 0.68 **^a^**	6.05 ± 0.61 **^a^**	6.09 ± 0.33 **^a^**	5.03 ± 0.63 **^ab^**	5.81 ± 0.48 **^a^**	4.93 ± 0.36 **^ab^**
TMA (ppm)	2.72 ± 0.21 **^c^**	2.76 ± 0.21 **^bc^**	2.96 ± 0.12 **^bc^**	2.56 ± 0.15 **^c^**	2.76 ± 0.39 **^bc^**	3.64 ± 0.26 **^a^**	3.65 ± 0.16 **^a^**	3.34 ± 0.05 **^ab^**	0.67 ± 0.16 **^d^**	2.47 ± 0.22 **^c^**	0.71 ± 0.04 **^d^**
TMAO (ppm)	250.67 ± 11.37 **^b^**	315.00 ± 21.17 **^ab^**	268.33± 41.28 **^ab^**	285.33 ± 18.50 **^ab^**	245.33 ± 34.99 **^b^**	297.00 ± 14.00 **^ab^**	340.67 ± 43.09 **^a^**	281.00 ± 25.71 **^ab^**	276.67 ± 18.72 **^ab^**	315.00 ± 30.61 **^ab^**	275.33 ± 37.07 **^ab^**

Values within the same row followed by different letters (a–d) are significantly different *p* < 0.05 (Tukey test).

**Table 2:** Abundance of the most potent odor-active compounds of HMH (x10^7^ Arbitrary Unit (U.A.)) after dissolution and after the different treatments (mean ± standard deviation).

	pH 7 after Dissolution	pH 7 -N -D	pH 7 -N +D	pH 7 +N -D	pH 7 +N +D	pH 7 -N +D + Alkalization pH 10	pH 10 after Dissolution	pH 10 -N -D	pH 10 -N +D	pH 10 +N -D	pH 10 +N +D
3-Methylbutanal	4.87 ± 0.72 ^a^	4.24 ± 0.90 ^abc^	3.16 ± 0.10 ^cde^	3.34 ± 0.27 ^bcde^	2.05 ± 0.08 ^e^	2.44 ± 0.38 ^de^	4.37 ± 0.64 ^abc^	4.46 ± 0.27 ^abc^	3.74 ± 0.05 ^abcd^	4.72 ± 0.53 ^a^	4.60 ± 0.22 ^ab^
2-Methylbutanal	2.57 ± 0.52 ^a^	1.47 ± 0.69 ^bc^	0.71 ± 0.06 ^cd^	0.74 ± 0.15 ^cd^	0.39 ± 0.05 ^d^	0.82 ± 0.14 ^cd^	2.24 ± 0.36 ^ab^	2.44 ± 0.19 ^ab^	1.67 ± 0.14 ^abc^	2.43 ± 0.58 ^ab^	2.14 ± 0.15 ^ab^
1-Methyl-1H-tetrazole	3.04 ± 0.64 ^a^	0.00 ± 0.00 ^b^	0.00 ± 0.00 ^b^	0.00 ± 0.00 ^b^	0.00 ± 0.00 ^b^	0.00 ± 0.00 ^b^	0.00 ± 0.00 ^b^	0.00 ± 0.00 ^b^	0.00 ± 0.00 ^b^	0.00 ± 0.00 ^b^	0.00 ± 0.00 ^b^
2,3-Pentanedione	1.63 ± 0.51 ^a^	1.70 ± 0.46 ^a^	1.24 ± 0.32 ^abc^	1.37 ± 0.04 ^ab^	1.40 ± 0.29 ^ab^	0.37 ± 0.13 ^bcd^	0.42 ± 0.20 ^bcd^	0.32 ± 0.01 ^bcd^	0.18 ± 0.01 ^d^	0.30 ± 0.09 ^cd^	0.22 ± 0.08 ^cd^
Pentanal	2.59 ± 0.51 ^a^	1.66 ± 0.51 ^b^	1.12 ± 0.29 ^bc^	1.26 ± 0.09 ^bc^	1.17 ± 0.28 ^bc^	0.55 ± 0.18 ^c^	1.49 ± 0.41 ^b^	1.48 ± 0.15 ^b^	1.05 ± 0.07 ^bc^	1.80 ± 0.40 ^ab^	1.59 ± 0.07 ^b^
Hexanal	2.86 ± 0.86 ^a^	2.47 ± 0.49 ^abc^	2.48 ± 0.56 ^ab^	2.66 ± 0.20 ^ab^	1.81 ± 0.14 ^abcd^	0.75 ± 0.31 ^d^	2.03 ± 0.39 ^abc^	1.58 ± 0.19 ^bcd^	1.26 ± 0.10 ^cd^	2.15 ± 0.52 ^abc^	2.02 ± 0.11 ^abc^
(Z)-4-Heptenal	2.89 ± 0.79 ^a^	2.80 ± 0.49 ^ab^	1.30 ± 0.53 ^bcd^	2.07 ± 0.24 ^abc^	1.27 ± 0.06 ^cd^	0.52 ± 0.31 ^d^	1.82 ± 0.23 ^abc^	1.39 ± 0.21 ^cd^	1.10 ± 0.16 ^cd^	1.93 ± 0.63 ^abc^	1.64 ± 0.16 ^bcd^
Heptanal	0.58 ± 0.38 ^ab^	0.56 ± 0.38 ^ab^	0.53 ± 0.22 ^ab^	0.77 ± 0.11 ^a^	0.52 ± 0.14 ^ab^	0.14 ± 0.10 ^b^	0.38 ± 0.10 ^ab^	0.48 ± 0.03 ^ab^	0.33 ± 0.09 ^ab^	0.43 ± 0.06 ^ab^	0.50 ± 0.09 ^ab^
Methional	0.053 ± 0.020 ^ab^	0.052 ± 0.023 ^ab^	0.039 ± 0.018 ^abc^	0.056 ± 0.013 ^ab^	0.072 ± 0.026 ^a^	0.009 ± 0.007 ^c^	0.016 ± 0.006 ^bc^	0.015 ± 0.001 ^bc^	0.015 ± 0.007 ^bc^	0.015 ± 0.008 ^bc^	0.015 ± 0.002 ^bc^
Benzaldehyde	4.22 ± 1.65 ^bc^	5.00 ± 1.30 ^abc^	2.32 ± 0.32 ^cd^	4.52 ± 0.20 ^abc^	2.57 ± 0.24 ^cd^	1.44 ± 0.71 ^d^	3.95 ± 0.50 ^bcd^	5.56 ± 0.72 ^ab^	4.86 ± 0.25 ^abc^	6.97 ± 1.83 ^a^	5.82 ± 0.22 ^ab^
(Z)-6-Octen-2-one	0.58 ± 0.24 ^cd^	0.80 ± 0.28 ^bc^	0.18 ± 0.08 ^d^	0.52 ± 0.03 ^cd^	0.085 ± 0.036 ^d^	0.40 ± 0.25 ^cd^	1.37 ± 0.23 ^ab^	1.29 ± 0.18 ^ab^	0.44 ± 0.15 ^cd^	1.51 ± 0.39 ^a^	0.62 ± 0.08 ^cd^
(E,E)-2,4-Heptadienal	0.15 ± 0.12 ^ab^	0.13 ± 0.02 ^ab^	0.37 ± 0.31 ^ab^	0.49 ± 0.35 ^a^	0.10 ± 0.01 ^ab^	0.00 ± 0.00 ^b^	0.015 ± 0.005 ^b^	0.028 ± 0.028 ^b^	0.00 ± 0.00 ^b^	0.015 ± 0.017 ^b^	0.019 ± 0.01 ^b^
Octanal	0.70 ± 0.38 ^a^	0.72 ± 0.12 ^a^	0.56 ± 0.23 ^ab^	0.72 ± 0.08 ^a^	0.53 ± 0.06 ^ab^	0.00 ± 0.00 ^c^	0.40 ± 0.20 ^abc^	0.17 ± 0.07 ^bc^	0.31 ± 0.03 ^abc^	0.63 ± 0.19 ^ab^	0.53 ± 0.07 ^ab^
2-Nonanone	0.73 ± 0.32 ^ab^	0.68 ± 0.21 ^abc^	0.15 ± 0.09 ^cd^	0.49 ± 0.13 ^abcd^	0.088 ± 0.024 ^d^	0.17 ± 0.10 ^cd^	0.97 ± 0.27 ^a^	0.93 ± 0.05 ^a^	0.19 ± 0.04 ^bcd^	0.99 ± 0.39 ^a^	0.28 ± 0.02 ^bcd^
(E,Z)-2,6-Nonadienal	0.35 ± 0.15 ^a^	0.45 ± 0.01 ^a^	0.47 ± 0.23 ^a^	0.36 ± 0.06 ^a^	0.41 ± 0.03 ^a^	0.032 ± 0.023 ^b^	0.065 ± 0.016 ^b^	0.045 ± 0.005 ^b^	0.047 ± 0.011 ^b^	0.074 ± 0.021 ^b^	0.070 ± 0.023 ^b^

Values within the same row followed by different letters (a–e) are significantly different *p* < 0.05 (Tukey test).

**Table 3:** S scores given by the twenty panel members for the five assessed conditions.

	pH 7 after Dissolution	pH 10 after Dissolution	pH 7 +N +D	pH 7 -N +D + Alkalization pH 10	pH 10 -N +D
S scores	80.0 ^a^	70.0 ^ab^	60.0 ^bc^	50 ^cd^	40.0 ^d^

Values within the same row followed by different letters (a–d) are significantly different *p* < 0.05 (Wilcoxon test).

## Data Availability

Not applicable.
